# Geographic Origin and Vertical Transmission of *Leishmania infantum* Parasites in Hunting Hounds, United States

**DOI:** 10.3201/eid2806.211746

**Published:** 2022-06

**Authors:** Susanne U. Franssen, Mandy J. Sanders, Matt Berriman, Christine A. Petersen, James A. Cotton

**Affiliations:** Ludwig-Maximilians-Universität Munich, Munich, Germany (S.U. Franssen);; Wellcome Sanger Institute, Hinxton, UK (S.U. Franssen, M.J. Sanders, M. Berriman, J.A. Cotton);; University of Iowa College of Public Health, Iowa City, Iowa, USA (C.A. Petersen);; Center for Emerging Infectious Diseases, University of Iowa, Iowa City (C.A. Petersen)

**Keywords:** leishmaniasis, *Leishmania infantum*, hunting hounds, vertical transmission, genomics, clonal evolution, parasites, United States

## Abstract

Vertical transmission of leishmaniasis is common but is difficult to study against the background of pervasive vector transmission. We present genomic data from dogs in the United States infected with *Leishmania infantum* parasites; these infections have persisted in the apparent absence of vector transmission. We demonstrate that these parasites were introduced from the Old World separately and more recently than *L. infantum* from South America. The parasite population shows unusual genetics consistent with a lack of meiosis: a high level of heterozygous sites shared across all isolates and no decrease in linkage with genomic distance between variants. Our data confirm that this parasite population has been evolving with little or no sexual reproduction. This demonstration of vertical transmission has profound implications for the population genetics of *Leishmania* parasites. When investigating transmission in complex natural settings, considering vertical transmission alongside vector transmission is vital.

Leishmaniasis is a disease caused by obligate intracellular protozoan parasites of the genus *Leishmania*, including *Leishmania infantum* (*1*). Zoonotic visceral leishmaniasis (ZVL) occurs in countries to which the disease is endemic and enzootic in human and animal populations. Dogs are the predominant domestic reservoir of ZVL and thus play a critical role in its ecology and control. Seropositivity is often evident in dogs before visceral leishmaniasis (VL) can be observed in humans (*2*), and dog ownership is a risk factor for human disease (*3*–*5*). As such, control measures in locations where ZVL is prominent include insecticide treatment or culling of dogs.

Although ZVL is transmitted primarily through phlebotomine sand flies (*6*), the role of other means of transmission, particularly vertical transmission, has been demonstrated (*7*–*10*). Transplacental transmission of *L. infantum* parasites can maintain infection within dog populations (*8*,*9*); pups have been shown to be infected in utero (*11*–*13*). Vertical transmission is not unique to dogs (*14*,*15*), and case reports have identified vertical transmission of VL as a cause of infant illness and death in humans (*16*,*17*). Beyond these reports, little is known about the risks of vertical transmission in dogs or humans. *Leishmania* parasites are thought to replicate exclusively clonally as intracellular amastigotes in vertebrate hosts. In contrast, in sand flies they undergo transformation into promastigotes, where they can still reproduce clonally but can also undergo meiosis to complete sexual reproduction (*18*,*19*), although sexual reproduction is not obligatory for transmission. Nothing is known about the transmission genetics of vertically transmitted *Leishmania* populations (*8*,*20*,*21*) or how the absence of vector stages affects the establishment or pathogenicity of mammalian infections.

In the United States, leishmaniasis is enzootic in hunting dogs. ZVL was first identified in 1980 in a dog with no travel outside of the United States. A large outbreak in 1999 prompted an investigation by the Centers for Disease Control and Prevention to determine the burden of disease in US hunting hounds (*22*,*23*). This investigation established the likely introduction of infected dogs from ZVL-endemic areas of Europe through the United Kingdom, but no testing of dogs outside the United States was performed, and genomic similarity to *L. infantum* parasites from Europe and South America was not evaluated (*23*,*24*).

We subsequently established the primary route of transmission as vertical from dam to pup (*9*,*25*). Despite extensive surveillance associated with these infected dogs (*26*,*27*), no naturally *L. infantum*–infected sand fly has been found in the United States. Although vector transmission of *L. infantum* parasites from these hunting dogs has been experimentally demonstrated (*27*,*28*), it does not appear to be involved in these natural infections.

We examined whole-genome sequences of *L. infantum* parasites from canine autochthonous infection within the United States and sought to identify a likely geographic origin. We looked for evidence of recombination between these *L. infantum* isolates to test for genomic evidence of predominantly vertical transmission. Many dogs are imported from ZVL-endemic areas to non–ZVL-endemic areas; our findings highlight the need for increasing awareness and testing before import of dogs from ZVL-endemic countries (*29*).

## Methods

### Ethics 

All dogs were enrolled with informed consent from their caretakers, and protocols followed were approved by the University of Iowa Institutional Animal Care and Use Committee. This AAALAC International–accredited institution follows the requirements for the US National Institutes of Health Office of Laboratory Animal Welfare Assurances and operates under the 2015 reprint of the Public Health service Policy on Humane Care and Use of Laboratory Animals.

### Sample Collection of Parasites from US Hunting Dogs

The 7 *L. infantum* samples from US hunting dogs used in this study were identified during a retrospective cohort study of *L. infantum* infection in US hunting dogs (*26*,*27*,*30*). To identify *Leishmania*-infected dogs, an active surveillance cohort of 4 large (>50 dogs each) kennels was established from 3 different states in the midwestern United States during 2007–2017. Licensed veterinarians collected 1–5 mL whole blood and serum samples from all dogs at these kennels. Dogs were considered infected if they were positive by quantitative PCR detecting *Leishmania*-specific DNA and had *Leishmania*-specific antibodies (*31*). Parasites from the buffy coat of *Leishmania*-positive dogs were cultured in both Schneider and HOMEM media overnight at 26°C then placed onto agar slants and incubated for 3–4 weeks and observed daily for growth. Parasite cultures include 1 sibling pair (foxymo_01, foxymo_02); remaining dogs all have different grandparents. Because of the frequent exchange of hunting dogs among kennels and states, within 2 generations the ancestors of the sampled dogs came from 12 kennels and 9 different US states (Georgia, Illinois, Iowa, Kansas, Minnesota, Missouri, New Jersey, New York, and Virginia) that included the primary US locations for hunting hound breeding.

### Whole-Genome Sequencing of Parasite DNA from Hunting Dogs

We used QIAamp DNA Blood Mini Kit (QIAGEN, https://www.giagen.com) according to manufacturer specifications to isolate DNA directly from primary parasite cultures. We thawed parasite cultures, counted, and placed 1 million parasites into Trizol Reagent (ThermoFisher Scientific, https://www.thermofisher.com) and extracted according to manufacturer specifications. We assessed quality and quantity of isolated DNA by using NanoDrop 2000 (ThermoFisher Scientific).

### DNA Sequencing

We sheared DNA into 400–600-bp fragments by focused ultrasonication using the Covaris Adaptive Focused Acoustics technology (Covaris, https://www.covaris.com). We performed 2 methods of DNA sequencing, depending on the amount of DNA supplied, by using the NEBNext DNA Library Prep kit (New England BioLabs, https://www.neb.com). For volumes <500 ng, we amplified libraries by using KAPA HiFI DNA polymerase (Kapa Biosystems, https://kapabiosystems.com) and generated 100-bp paired-end reads on the Illumina HiSeq 2000 (Illumina, https://www.illumina.com). For volumes >500 ng, we generated amplification-free libraries and obtained 150-bp paired-end reads on the Illumina HiSeq X10 (Illumina). We performed sequencing following manufacturers’ standard protocols.

### Genomic Analysis Pipeline

We analyzed the genomic data of 7 *L. infantum* US hound isolates with an additional 92 publicly available *L. infantum* isolates sampled from a global distribution (Appendix 1). For all samples, we subjected newly generated and downloaded fastq files to identical analysis pipelines. We trimmed reads using Trimmomatic version 0.39 (http://www.usadellab.org/cms/?page=trimmomatic) (parameters “ILLUMINACLIP:PE_adaptors.fa:2:30:10 TRAILING:15 SLIDINGWINDOW:4:15 MINLEN:50”) and mapped them against the reference genome of JPCM5 v45 (https://tritrypdb.org) with BWA version 0.7.17 (bwa mem -M option) (*32*). Single-nucleotide polymorphisms (SNPs) were called using GATK version 4.1.2.0 (*33*): HaplotypeCaller was used with parameters “-ERC GVCF–annotate-with-num-discovered-alleles–sample-ploidy 2” to generate gvcf files for each sample, then combined using “GenomicsDBImport” and genotyped with “GenotypeGVCFs.” Calls were filtered with “VariantFiltration” (filters: “QD<2.0, MQ<50.0, FS>20.0, SOR>2.5, BaseQRankSum<-3.1, ClippingRankSum<-3.1, MQRankSum<-3.1, ReadPosRankSum<-3.1and DP<6”) and only polymorphic SNPs retained. We removed SNPs with >20% missing calls across samples, reducing the total number of SNPs from 43,528 to 43,336.

### Phylogenetic Reconstruction and Admixture Analysis

We performed phylogenetic reconstruction by using distance-based and maximum-likelihood methods on genome-wide genotype calls. For the distance-based approach, we calculated pairwise Nei D distances and reconstructed trees by the neighbor-joining method using the R packages StAMPP version 1.6.1 (*34*) and ape version 5.4. We based bootstrap values on 100 replicates. For maximum-likelihood phylogenies, we converted the vcf file to fasta format with IUPAC codes using bcftools consensus. We estimated 1,000-bootstrap maximum-likelihood phylogenies by using RAxML-NG version 0.8.1-c1 (*35*) and the GTJC model that captures changes between heterozygous and homozygous states.

**Figure 1:**
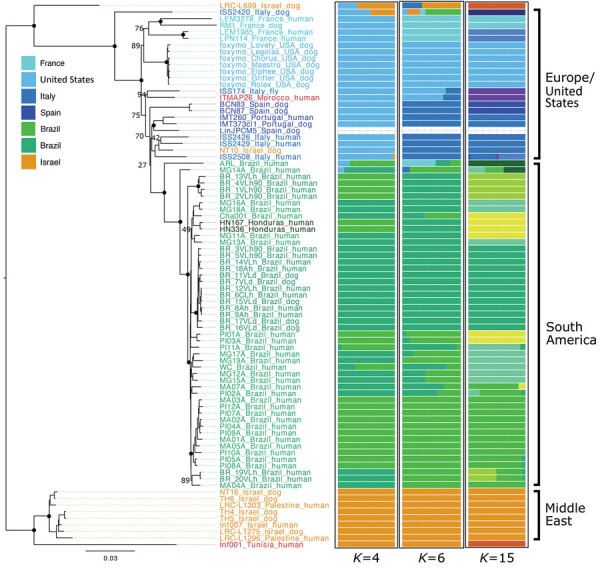
Neighbor-joining tree based on pairwise Nei distances demonstrating geographic origin of US hound *Leishmania* isolates. Phylogenies were reconstructed on the basis of whole-genome genotype calls of 83 parasite samples representing the dominant *L. infantum* zymodeme MON-1 from the United States, Europe, South America, and the Middle East, which were the samples most relevant in the context of the origin of the US samples (Appendix 2 Figure 2). The 3 righthand columns indicate population grouping using admixture with best fitting total number of groups (Appendix 2 Figure 1, panel A).

We preprocessed genome-wide SNPs for admixture analysis version 1.3.0 (*36*) only with plink version 1.90 changing the vcf format into ped and map format and removing SNPs with a missing fraction of >0.05 and variants closer to each other than 2,000-bp with the arguments “–geno” and “–bp-space.” We ran admixture for values of *K* from 1 to 20 and optimal numbers of groups (*K*) were chosen on the basis of lowest cross-validation error (Appendix 2 Figure 1). Because there was no clear number of *K* at which the cross-validation error plateaued, we present analyses with the smallest *K* at first sign of plateauing of the error and 2 larger *K*s with smaller errors.

### Molecular Clock Dating

We used 2 molecular clock approaches. The first method was a simple clock model using PATHd8 (*37*) for all RAxML-NG bootstrap trees, constraining the root of the non-US New World clade to 537 years ago. The second method was a Bayesian approach that used BEAST version 1.10.4 (https://beast.community) to enable flexible modeling of rate variation with standard substitution models, a narrow uniform prior of 536.9–537.1 years for the New World clade and leaf heights set to the year of collection (Appendix 1), or constrained to 2005–2007 for samples from (*39*) and to 1900–2020 for the sample ‘DOG_STRAIN’ of unknown sampling date (*38*). New World and US hound clades were constrained to be monophyletic, and Bayesian Markov Chain Monte Carlo analysis was initialized with the RAxML-NG phylogeny for concatenated chromosomes. The substitution model was Hasegawa-Kishino-Yano with a 4-category gamma distribution of rate variation across sites. Results are based on 8 independent Bayesian Markov Chain Monte Carlo chains of 10 million generations, 1 million generations burn-in, and convergence checked using Tracer version 1.7.1 (https://beast.community/tracer). We accepted analyses if 6 out of 8 chains were at similar likelihoods for 2 million generations. Remaining parameters were defaults from Beauti version 1.10.4. Only results for both strict and uncorrelated gamma-distributed clocks converged and are shown.

### Population Genomics Analysis

We grouped parasite samples according to geographic origin and isolated host type (Table). Groups were characterized by their number of segregating SNPs, inbreeding coefficients, and linkage decay with distance. We performed analysis in R (R Foundation for Statistical Computing, https://www.r-project.org) with the exception of R^2^ estimates, which we estimated as genotype correlations with vcftools version 0.1.16 (*41*) and parameters “–geno-r2” and “–interchrom-geno-r2.” We used genotype correlations because haplotypes cannot be accurately phased for our small population sets. We calculated the inbreeding coefficient F based on the formula *F* = 1 –((c_AB_/N)/(2 × f_A_ × f_B_)), where c_AB_ represents the heterozygote count, N the group size, and f_A_ and f_B_ the frequency of alleles A and B.

**Table:** Summary of groups compared in analysis of geographic origin and vertical transmission of *Leishmania infantum* in hunting hounds, United States*

Group name	Sample size	Sample names	Location	Isolation year	Time span of isolations, y	Host	Disease phenotype	Source
US_d	7	foxymo_01, foxymo_02, foxymo_03, foxymo_04, foxymo_05, foxymo_06, foxymo_07	Midwestern United States	2009–2016	8	Dog	CanL	This study
BR_d	5	BR_7VLd, BR_11VLd, BR_15VLd, BR_16VLd, BR_17VLd	Rio Grande do Norte, Brazil	2010–2012	3	Dog	VL	(*43*)
IS_d	5	NT16, TH4, TH5, TH6, LRC-L1275	Israel	2005–2012	8	Dog	Unknown	(*38*)
BR_RGN_VLh	5	BR_1VLh90, BR_2VLh90, BR_3VLh90, BR_4VLh90, BR_5VLh90	Rio Grande do Norte, Brazil	1991–1993	3	Human	VL	(*43*)
BR_RGN_VLhAh	6	BR_12VLh, BR_14VLh, BR_19VLh, BR_8Ah, BR_9Ah, BR_18Ah	Rio Grande do Norte, Brazil	2011–2013	4	Human	VL or asymptomatic	(*43*)
BR_MA_VLh	6	MA01A, MA02A, MA03A, MA04A, MA05A, MA07A	Maranhão, Brazil	2005–2006	2	Human	VL	(*39*)
BR_MG_VLh	9	MG11A, MG12A, MG13A, MG14A, MG15A, MG16A, MG17A, MG18A, MG19A	Minas Gerais, Brazil	2005	1	Human	VL	(*39*)
BR_PI_VLh	11	PI01A, PI02A, PI03A, PI04A, PI05A, PI07A, PI08A, PI09A, PI10A, PI11A, PI12A	Piauí, Brazil	2005–2006	2	Human	VL	(*39*)
CH_mix	7	D_2, Peking, DOG_STRAIN, RACOON_DOG, SKIN, STRAIN_A, STRAIN_B	China	1954–1983	30	Human, dog, raccoon dog	VL, unknown	(*38*)
FR_mix	4	LEM1985, LEM3278, LPN114, RM1	France	1987–1996	10	Human, dog	CanL, unknown	(*38*)
IP_mix†	7	**NT16**, **TH4**, **TH5**, **TH6**, **LRC-L1275**, LRC-L1296, LRC-L1303	Israel/ Palestine	2005–2012	8	Human, dog	Unknown	(*38*)
IT_mix	5	ISS174, ISS2420, ISS2426, ISS2429, ISS2508	Italy	1985–2002	18	Human, dog, sand fly	VL, CanL, sand fly	(*38*)
SP_mix‡	5	LinJPCM5, BCN83, BCN87, IMT373cl1, IMT260	Spain/ Portugal	1987–2005	19	Human, dog	CL, VL, unknown	(*38**, **40*),

*Samples and corresponding groups were chosen from the total of 99 isolates (Appendix 2 Figure 2) to represent geographic regions or countries with at least 5 samples available and a focus on groups with dog isolates only, humans only, and a mixture of hosts for comparison. †Samples in groups IS_d are also part of group IP_mix and are indicated in bold. ‡The group SP_mix contains only isolates from Spain and Portugal that are in the clade of the known including several known MON-1 samples. The isolates Inf055, Inf004 from the non–MON-1 clade are not included.

### Aneuploidy Estimation

We estimated sequencing coverage on the basis of sample-specific mapped bam files. For each sample, indels were determined and indel realignment was performed with the GATK version 3.6 (*33*) tools “RealignerTargetCreator” and “IndelRealigner.” Quality filtering and duplicate removal was done with samtools version 1.3 using the parameters “-F 1024 -f 0x0002 -F 0x0004 -F 0x0008.” Coverage was estimated with bedtools version 2.17.0 (*42*) genomecov and parameters “-d -split.” For each sample, the median coverage per chromosome was assumed to represent the diploid state, so chromosome somy = (chromosome_coverage/median_coverage) × 2. Allele frequencies for isolate-specific SNPs were estimated on the basis of previous bam files and quality filtered with samtools “-q 20 -f 0x0002 -F 0x0004 -F 0x0008.” Coverage by genomic position was obtained with samtools mpileup “-d 3500 -B -Q 10” and transformed into sync format with mpileup2sync “–min-qual 20” (*43*).

## Results

### Independent Introduction of US Hound–Derived Parasites from the Mediterranean Region

To assess the geographic origins of *L. infantum* parasites within US hunting dogs, we generated whole-genome sequence data for 7 *L. infantum* isolates from outbred hounds from 4 kennels in the midwestern United States and an ancestry tracing back to kennels in 9 US states within 2 generations with haploid coverage ranging from 29 to 78 (median 69). We compared these samples with 92 previously published *L. infantum* genome sequences of other strains from other global populations (*38*,*39*,*44*) (Appendix 1).

We constructed distance-based and ML phylogenies from whole-genome SNP variants to compare *L. infantum* genomes from US dogs to samples from *L. infantum*–endemic regions of South America and the Old World. Parasites from US hounds were monophyletic, part of the *L. infantum* MON-1 clade (*38*,*45*), and clearly distinct from *L. infantum* isolates from South America (Figure 1; Appendix 2 Figure 2). These factors suggest independent introduction to the New World. The genetically closest parasite samples were from southern Europe, but the exact origin was ambiguous. Distance-based methods suggested 4 samples from France as genetically most closely related to US isolates (Figure 1; Figure 2, panel A). The ML phylogeny placed US parasites close to a more widespread group of MON-1 parasites (Figure 2, panel B).

**Figure 2:**
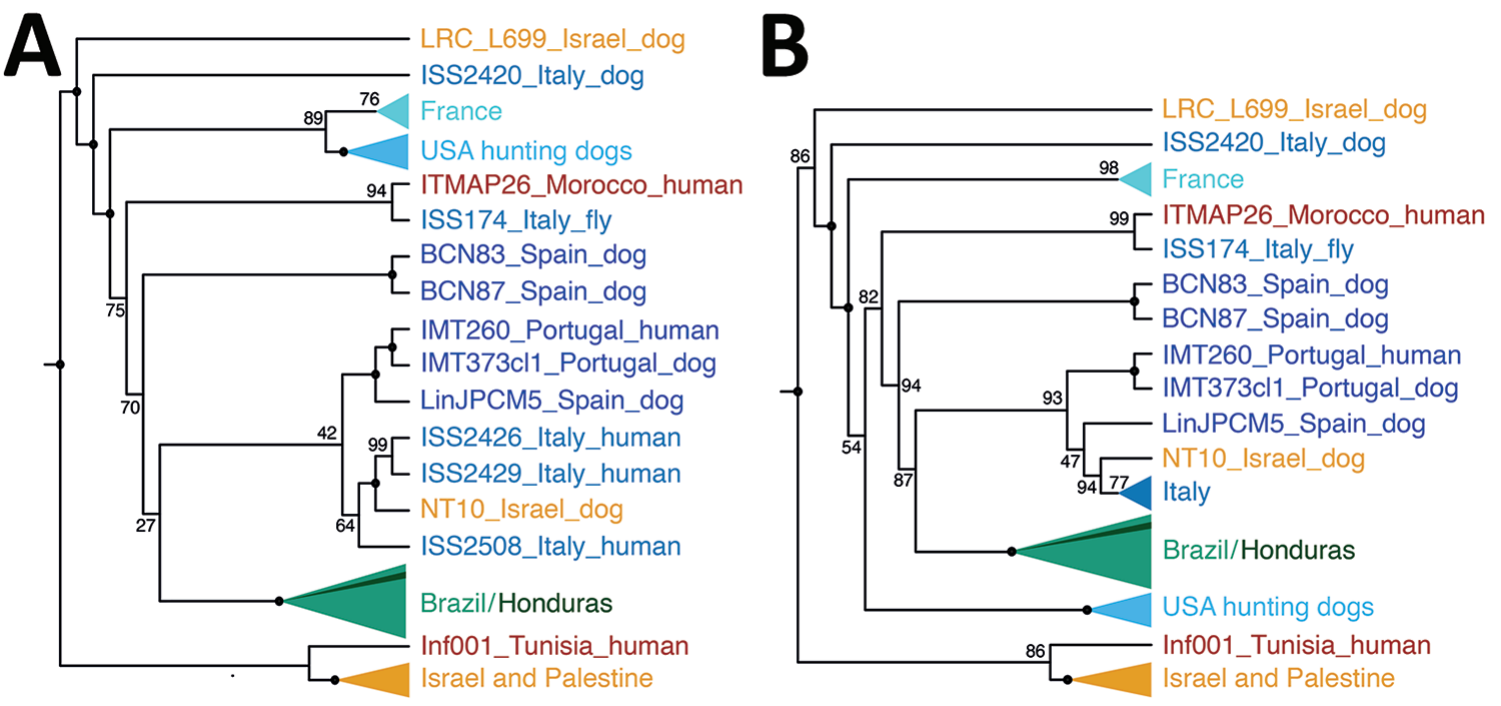
Geographic origin of US hound *Leishmania* isolates. A) Cladogram of the neighbor-joining tree from Figure 1 showing monophyletic groups for better visibility of evolutionary relationships of the US hound parasites. B) Cladogram of the maximum-likelihood phylogeny (Appendix 2 Figure 2, panel B). Cladograms were reconstructed on the basis of whole-genome genotype calls of 83 parasite samples representing the dominant *L. infantum* zymodeme MON-1 from the United States, Europe, South America, and the Middle East, which were the samples most relevant in the context of the origin of the US samples (Appendix 2 Figure 2). Numbers at internal nodes show bootstrap values.

To further investigate parasites’ relatedness, we performed admixture analysis, which was consistent with the phylogenetic results. We applied cross validation, a standard approach in admixture to determine an optimal number of populations (*K*) that best explains the relatedness between samples. Because this process did not identify a single optimal *K* (Appendix 2 Figure 1), we considered more than one *K* (Figure 1; Appendix 2 Figure 2). We concentrated our analysis on 83 core samples consisting of samples from the United States and other samples from the MON-1 clade (Figures 1, 2). For *K* = 4 populations, US hound parasites were placed together with all remaining samples from Europe and single samples from Israel and Morocco (Figure 1). For *K* = 6 and *K* = 15, US samples formed a separate group, only inferred to share ancestry with one sample from Italy and one from Morocco for *K* = 6. A similar pattern was present within the total set of 99 samples. For *K* = 7, US and 2 parasites from France grouped together, and for *K* = 11, US samples only shared substantial variation with 1 sample from Italy (Appendix 2 Figure 2, panel A), which together suggested a clear origin from Mediterranean Europe but no clear country of origin.

### Molecular Clock Dating Confirms Recent Divergence of US Hound–Derived Parasites

**Figure 3:**
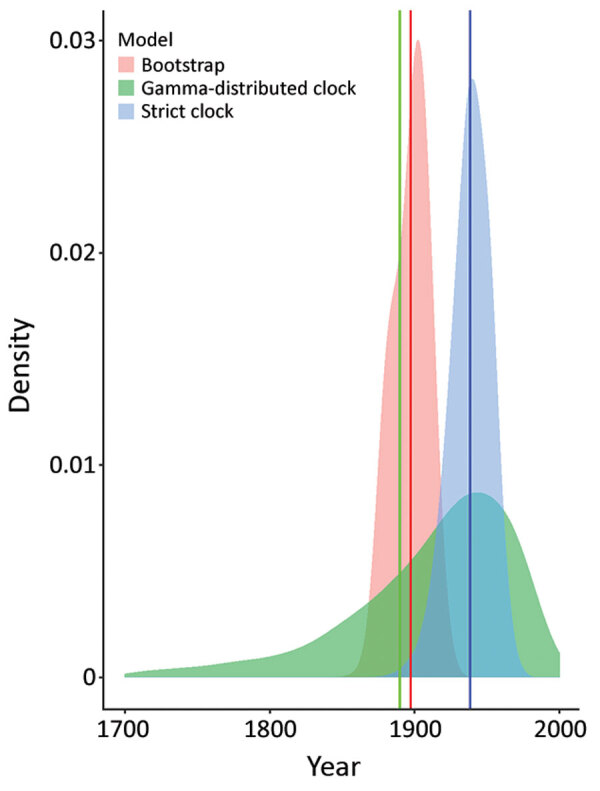
Molecular clock estimates of the date of the most recent common ancestor of US hound *Leishmania* samples. Shaded densities are normal kernel densities for the bootstrap estimates from PATHd8 analysis and from posterior samples for strict clock and relaxed clock with uncorrelated gamma-distributed rates in BEAST version 1.10.4 (https://beast.community). These distributions in each case represent the estimated uncertainty in the divergence date of *Leishmania infantum* isolates from US hounds and from Europe. Vertical lines in the same colors are at the means of each distribution.

We dated the independent introduction of US hound parasites by using 2 different molecular clock approaches, relying on previously estimated introduction of *L. infantum* parasites into the New World ≈500 years ago (*46*). The first analysis using our maximum-likelihood phylogeny estimated the mean date of divergence between US parasites and relatives from Europe as 1897 (95% CI 1873–1917), whereas 2 Bayesian approaches produced estimates of 1938 (strict clock, 95% highest posterior density CI 1910–1965) and 1889 (relaxed clock, 95% CI 1689–1991) (Figure 3). Estimates across a range of approaches thus suggest that US hound parasites were introduced much more recently than *L. infantum* parasites were introduced to South America.

### Patterns of Heterozygosity in US Hound Parasites Suggest Clonal Evolution

**Figure 4:**
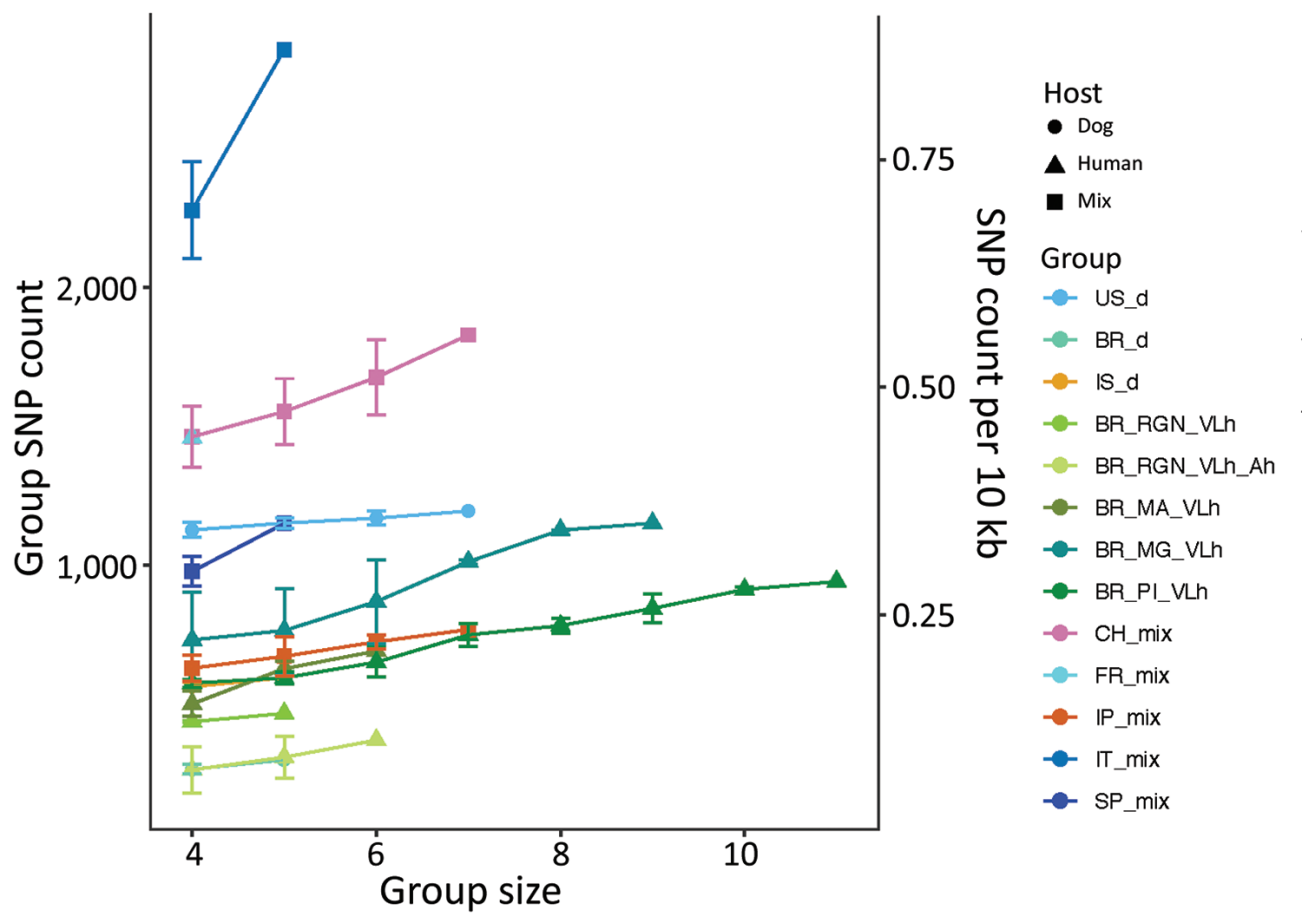
Number and density of segregating SNPs in each group of *Leishmania infantum* isolates by geographic region and type of host. Values are shown as both the number (left y-axis) and density (right y-axis) of segregating SNP sites in each group. Because group sizes vary, groups were subsampled in triplicate for each group size from 4 up to their respective size; means and SDs are shown. SNP, single-nucleotide polymorphism.

The genetic variation in a population should reflect its reproductive biology. We thus compared variation in US hound parasites with *L. infantum* populations isolated from dogs in areas where vector transmission occurs and with populations isolated from humans or a mixture of both hosts in other parts of the world (Table). Within-population diversity of the US hound parasites was intermediate between the high diversity of populations from the Old World and the low diversity of parasites from different regions within Brazil (Figure 4). For most populations, the number of polymorphic sites increased with sample size, indicating that increasing numbers of rare variants were detected with larger sample sizes. This sample size–based increase was minimal in the US hound parasite population, suggesting a large proportion of shared variation among these isolates.

**Figure 5:**
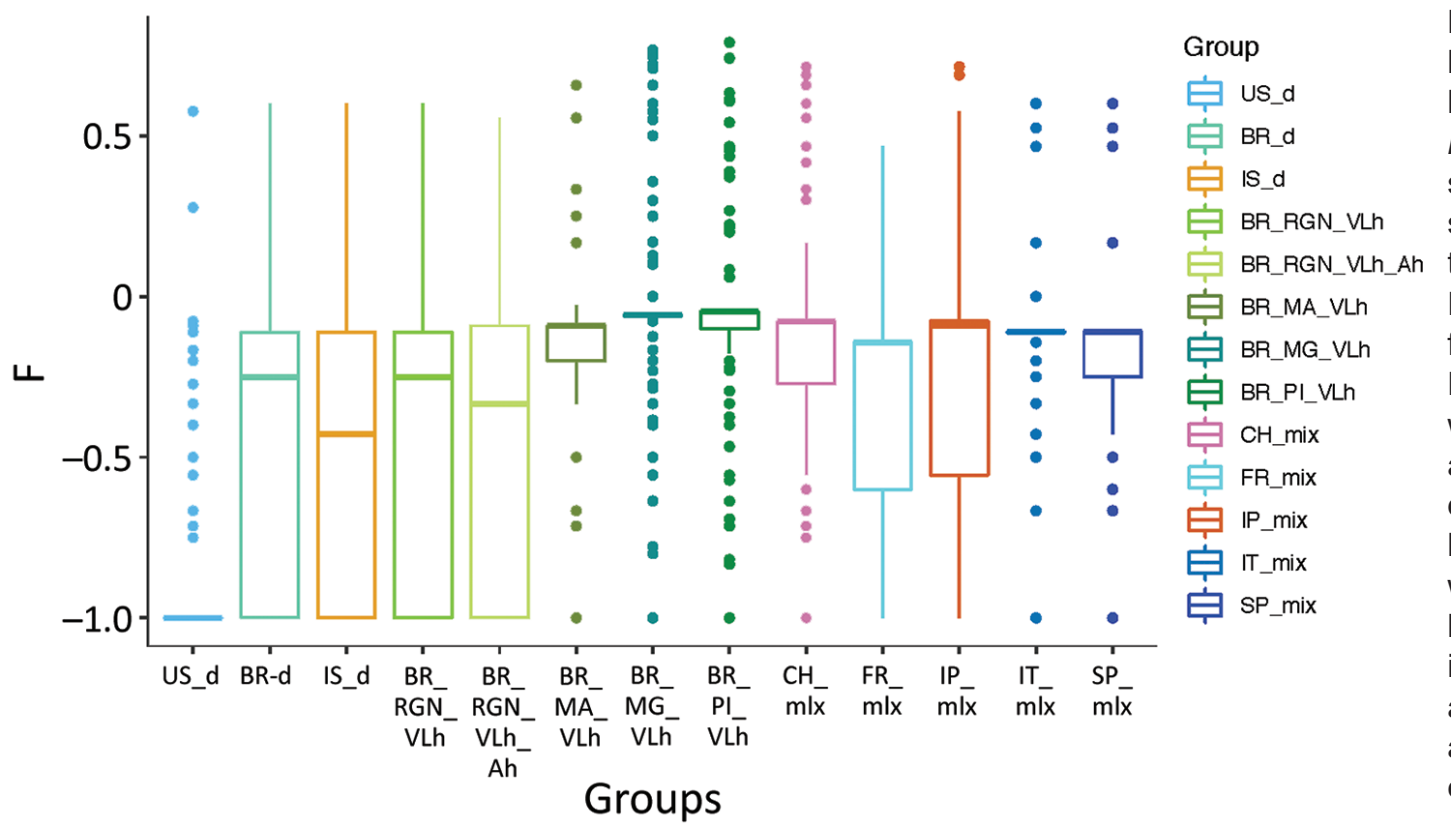
Extreme excess of heterozygous sites in the US hound–derived *Leishmania infantum* isolates. The group-specific inbreeding coefficient F is shown for all polymorphic sites in the respective parasite population. F measures the deviation of the frequency of heterozygotes from Hardy-Weinberg equilibrium with negative values indicating an excess and positive values a deficiency of heterozygotes over homozygotes. Horizontal lines within boxes indicate medians; box top and bottom lines indicate 25 and 75 percentiles; and error bars indicate minimum and maximum values, excluding outliers.

To explore this shared variation further, we directly estimated population heterozygosity through the inbreeding coefficient F and the fraction of population-specific polymorphic heterozygous SNP sites (Figure 5; Appendix 2 Figure 3). The inbreeding coefficient was significantly different between populations (Kruskal-Wallis test, χ^2^ = 2843.1, df = 12; p<0.001), and the US hound parasite population had exceptionally low F values compared with all other populations (Dunn test, adjusted; p<0.001) (Figure 5). This difference was largely caused by 79% of all polymorphic sites within US hound–derived parasites sharing the same heterozygous genotype across all 7 sampled hound isolates. This extreme excess of shared heterozygosity is present across all chromosomes and is in strong contrast to the remaining populations. Absolute numbers of heterozygous sites in the US samples were higher than in other populations (Table; Appendix 2 Figure 4, panel A). This difference could be caused by either the accumulation of mutations during a period of clonal evolution shared by these samples or a hybrid origin of the founder strain of our US samples between 2 closely related *L. infantum* populations (Appendix 2 Figure 4, panel B), because clonal propagation would maintain any heterozygosity.

### No Evidence for Sexual Reproduction in *L. infantum* Isolated from US Hounds

**Figure 6:**
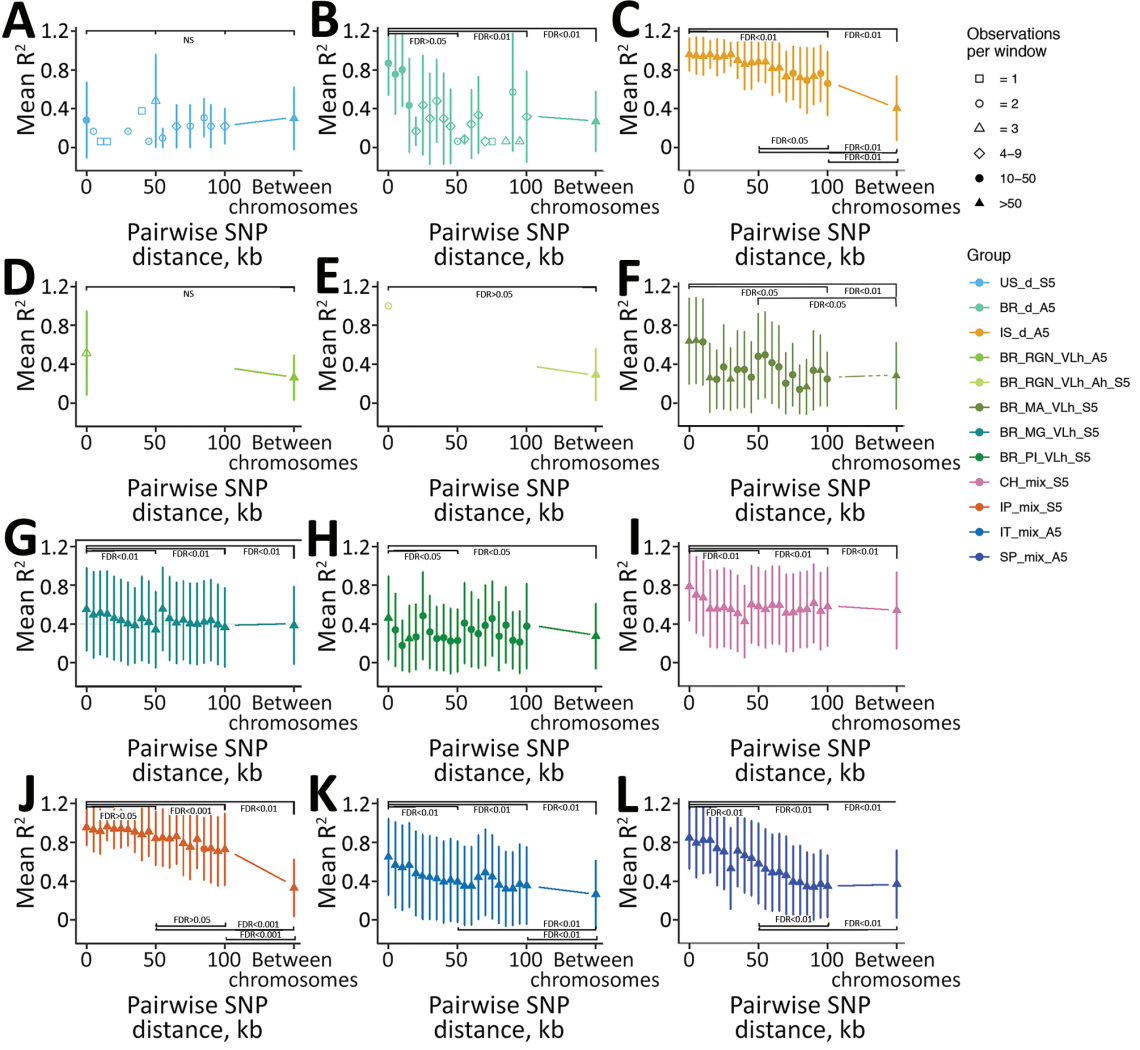
Decay of linkage disequilibrium with genomic distance across geographically confined groups of *Leishmania infantum* isolates. A) US_d_S5, B) BR_d_A5, C) IS_d_A5, D) BR_RGN_VLh_A5, E) BR_RGN_VLh_Ah_S5, F) BR_MA_VLh_Ah_S5, G) BR_MG_VLh_S5. H) BR_PI_VLh_S5, I) CH_mix_S5, J) IP_mix_A5, K) IT_mix_A5, L) SP_mix_A5. Long-range linkage disequilibrium was measured as R^2^ for pairs of SNPs up to 100 kb apart within chromosomes and located on different chromosomes. Symbols show mean R^2^ across SNP-pairs on all chromosomes, and lines show 1 SD for variants in bins of 5kb distance starting at the indicated distance. For groups with >5 samples, 5 have been randomly chosen to calculate R^2^ values, indicated in group names for each subplot (S6, subsampled 5; A5, all 5 samples of the group were used). Symbol shapes indicates the number of pairwise comparisons available for each distance bin. Statistical significance of comparisons between R^2^ between 4 different 5 kb windows at 0–4999 bp, 50–54.999 kb, 100–104.999 kb between SNP pairs for all between-chromosome comparisons are shown. FDR was determined based on the Kruskal-Wallis test, followed by the Dunn post hoc test when significant. For the groups in which only data for 2 of the 4 windows was present, the Mann-Whitney-Wilcoxon test was used. FDR, false discovery rate; NS, not significant; SNP, single-nucleotide polymorphism.

If *L. infantum* parasite transmission in US hunting dogs occurs solely through vertical transmission, we would expect genomic signatures of sexual reproduction to be absent because sexual reproduction is thought to be limited to the vector stage (*18*). Sexual reproduction returns proportions of heterozygous and homozygous variants to the Hardy-Weinberg equilibrium. We propose that the observed extreme excess of shared heterozygous sites in US hound parasites is possible because these parasites evolve clonally for many generations with no mechanism to reduce the number of heterozygous sites through sexual reproduction. To test this proposition, we investigated whether genetic linkage between pairs of SNPs reduces as the distance between loci increases, which would be expected if recombination is occurring. Almost all global *L. infantum* populations showed this expected decay in linkage within chromosomes, except US hound–derived parasites and 2 populations from Brazil (Figure 6). The 2 populations from Brazil had too few polymorphic sites to reliably assess linkage patterns. The US hound parasites also had relatively few sites for analysis, because unphased shared heterozygous sites cannot be used for linkage estimation. However, the remaining loci showed no evidence of linkage decay with genetic distance. Pairs of variants on different chromosomes showed very similar linkage to within-chromosome comparisons (Figure 6). This finding indicates that evidence for meiotic recombination in the US dog *L. infantum* population is lacking.

### Reduced Variation in Aneuploidy in Mammalian Host–Derived Parasites

**Figure 7:**
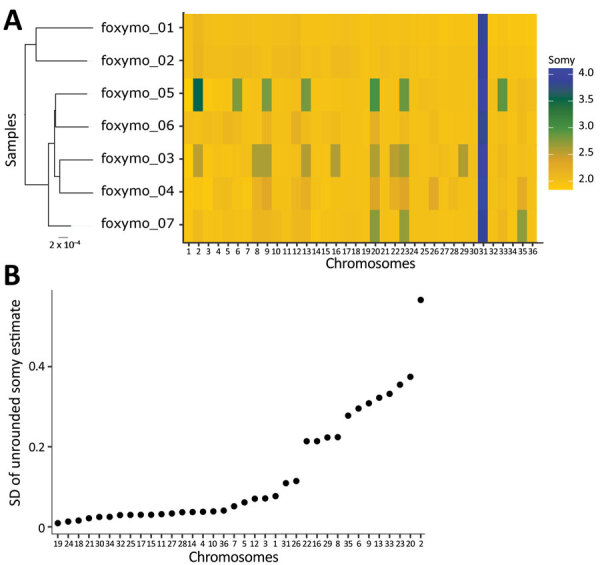
Aneuploidy variation of *Leishmania* isolates from US hunting hounds. A) Aneuploidy profiles, shown as a heatmap of estimated somy for each isolate and chromosome. The sample phylogeny is extracted from Figure 1. B) Chromosome-specific variation in somy across US hound isolates. Variation in somy between isolates provides a conservative estimate of somy variation, as it ignores within-isolate variation.

*Leishmania* populations frequently show variation in copy number of individual chromosomes with frequent aneuploidy turnover even within a clonal population (mosaic aneuploidy). Aneuploidy variation between US isolates was largely limited to one third of the chromosomes and variation did not correlate to chromosome-specific heterozygosity, which should have been reduced if aneuploidy turnover was high (Figure 7; Appendix 2 Figure 5). Although this estimate of aneuploidy variation through mean ploidy profiles between isolates is conservative, it supports initial findings that aneuploidy turnover might be greater in cultured promastigotes versus intra-host amastigotes (*47*,*48*).

## Discussion

Our data confirm that *L. infantum* found in US hounds represents an independent introduction of *Leishmania* into the New World. Although we cannot be definitive about the precise origin of US hound *L. infantum* isolates, they form part of the MON-1 clade, associated with canine leishmaniasis throughout the Mediterranean region. Closely related MON-1 samples are from Mediterranean Europe, consistent with epidemiologic findings that deer hunting hounds imported from France may have introduced *L. infantum* parasites into the US hound population, potentially through UK breeding connections (*29*).

Molecular clock analyses suggested that US hound parasites diverged from other *L. infantum* isolates around 1900, but parasitized dogs could have entered the United States more recently. These date estimates also depend on the assumed origin of the main New World subspecies (*L. infantum* subspecies *chagasi*) 537 years ago, the central estimate from an analysis of microsatellite data, although with very wide CIs (*46*). The safest interpretation of our analysis is therefore a much more recent divergence of US canine parasites from parasites in Europe than the main New World clade of *L. infantum* subsp. *chagasi*.

Our data confirmed the highly unusual genetics of the *L. infantum* population in US hounds. This parasite population demonstrated an excess of shared heterozygous loci, which could have been initiated by an already heterozygous founder strain. However, the preservation of heterozygous sites across our US samples is consistent with clonal reproduction, which is also confirmed by the absence of any signature of reduction in genetic linkage with genomic distance in this population. Without a broader sampling of parasites from US hounds, we cannot rule out that transmission via sand flies is occurring elsewhere in the United States. Similarly, we cannot quantify the amount of parasite sexual reproduction from these data and so cannot completely rule out that sexual reproduction and therefore vector transmission are occurring. However, our results are consistent with parasites replicating only clonally as amastigotes in dog phagocytes in the absence of sand fly vectors. No sand fly transmission of *L. infantum* parasites from dogs in the United States has been demonstrated (*7*,*9*,*28*), so we suspect that transmission within this population is largely occurring vertically and directly between dogs.

The population genetic signatures of vertical transmission we have found could be useful in characterizing the epidemiology of other *Leishmania* populations. The extent to which these signatures occur in more complex situations, such as with multiple introductions of parasites or mixed vertical and horizontal transmission, remains to be established. The most direct evidence of vertical transmission would be to find that the relatedness between parasite isolates directly reflected the pedigrees of the sampled dogs, although this would be potentially complicated by horizontal transmission between dogs (e.g., through blood-blood contact during fights) (*49*). Although we have not attempted to test this possibility, parasites from the pair of siblings included here (foxymo_01 and foxymo_02) were genetically closest to each other and clearly separated from all others.

In conclusion, our data confirm the 1999–2000 outbreak investigation finding by the Centers for Disease Control and Prevention that at least 1 *L. infantum* population in US dogs was a recent introduction from Europe, distinct and much more recent than the main population of *L. infantum* in South America. This population has reproduced largely or exclusively clonally, presumably as amastigotes within canine hosts. We see no evidence of recent recombination associated with vector transmission up to the limits of our detection levels; thus, transmission has likely occurred either vertically through maternal-offspring transplacental transmission or horizontally through blood-blood contact. The absence of evidence for vector-based transmission in the northern United States makes this an unusual, and perhaps unique, ecologic system. Our findings enable the study of many aspects of *Leishmania* biology without the complication of occasional vector transmission, including adaptation of parasites to the mammal host without the additional selection pressure of vector transmissibility, mutation rates, and rates of amastigote cell division.

Appendix 1 Additional data used in study of geographic origin and vertical transmission of *Leishmania infantum* parasites in hunting hounds, United States

Appendix 2 Additional information about geographic origin and vertical transmission of *Leishmania infantum* parasites in hunting hounds, United States
