# Supplementary material for: Geographic Origin and Vertical Transmission of Leishmania infantum Parasites in Hunting Hounds, United States
Source: Emerg Infect Dis. 2022 Jun;28(6):1211–23. doi: 10.3201/eid2806.211746 (PMC9155895; doi:10.3201/eid2806.211746)
Supplement: Appendix 2 — Additional information about geographic origin and vertical transmission of Leishmania infantum parasites in hunting hounds, United States [file 21-1746-Techapp-s2.pdf]

Article DOI: https://doi.org/10.3201/eid2806.211746

Geographic Origin and Vertical Transmission of *Leishmania infantum* Parasites in Hunting Hounds, United States

Appendix 2

Reduced Variation in Aneuploidy in Mammalian-Host Derived Parasites

Variation in copy number of individual chromosomes has been shown to occur very rapidly during clonal reproduction in vitro, leading to so-called mosaic aneuploidy, in which cells within a single clonal population have different aneuploidy profiles. In contrast, the little data available from experimental infections suggest this mosaicism may be less common in the intracellular amastigote stage present in mammals (*1,2*). The absence of sexual reproduction or its strong reduction in US hounds offered an opportunity to study variation in chromosome copy number in *Leishmania* amastigotes in a natural system.

We observed that most chromosomes were disomic in most US hound isolates. There was no association with the corresponding somy variability per chromosome and either the inbreeding coefficient or fraction of population-wide heterozygous sites. This would have been expected if somy reduction was frequently acting to reduce heterozygosity of individual cells (Appendix 2 Figure 5). However, there was some variation in somy levels (Figure 7, https://wwwnc.cdc.gov/EID/article/28/6/21-1746-F7.htm): most interestingly three isolates (foxymo_03, foxymo_05 and foxymo_07) showed increased somy levels for a largely shared set of chromosomes (Figure 7, panel A). As this somy-sharing is inconsistent with the relatedness of the isolates based on SNP data, it might indicate convergent aneuploidy changes in the dog hosts.

To confirm these somy estimates and that the shared heterozygosity in the hound isolates represent genuine heterozygous variants rather than homozygous variants between different cell populations within an isolate, we inspected allele frequency profiles by isolate. In the heterozygote case allele frequencies in the cell pool should be distributed at the frequency expected by the predicted somy (e.g. 0.5 for diploid and 0.33 and 0.66 for tetraploid

chromosomes). In most cases the allele frequencies match those expected from chromosome somies (Appendix 2 Figure 6), emphasizing that the remarkable heterozygosity found in US hound *L. infantum* samples could not be explained by different homozygous variants segregating within a parasite isolate.

References

1. Franssen SU, Takele Y, Adem E, Sanders MJ, Müller I, Kropf P, et al. Diversity and within-host evolution of *Leishmania donovani* from visceral leishmaniasis patients with and without HIV coinfection in northern Ethiopia. MBio. 2021;12:e0097121. PubMed https://doi.org/10.1128/mBio.00971-21

2. Franssen SU, Durrant C, Stark O, Moser B, Downing T, Imamura H, et al. Global genome diversity of the *Leishmania donovani* complex. eLife. 2020;9:e51243. PubMed https://doi.org/10.7554/eLife.51243

3. Dumetz F, Imamura H, Sanders M, Seblova V, Myskova J, Pescher P, et al. Modulation of aneuploidy in *Leishmania donovani* during adaptation to different in vitro and in vivo environments and its impact on gene expression. MBio. 2017;8:e00599–17. PubMed https://doi.org/10.1128/mBio.00599-17

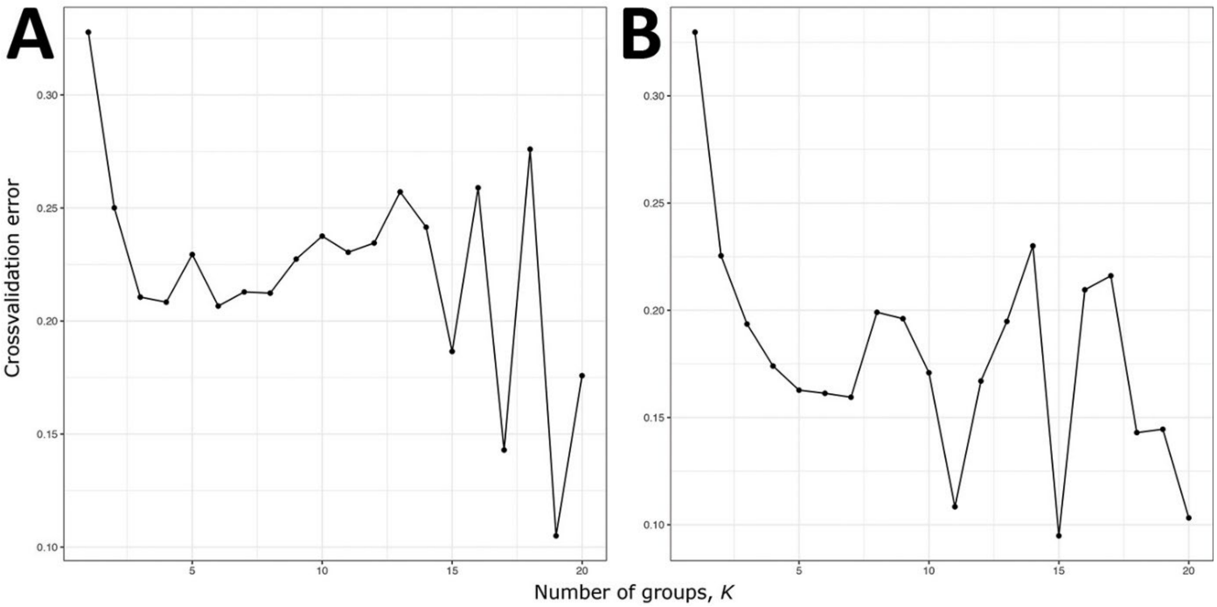

Appendix 2 Figure 1. Identification of optimal number of groups *K* for admixture analysis. The cross-validation error is shown for admixture analysis for values of *K* ranging from 1 to 20. Results for the admixture analysis with A) 83 core (Figure 1 in main article, https://wwwnc.cdc.gov/EID/article/28/6/21-1746-F1.htm) and B) 99 broader context (Appendix 2 Figure 2, panel A) *L. infantum* isolates are shown.

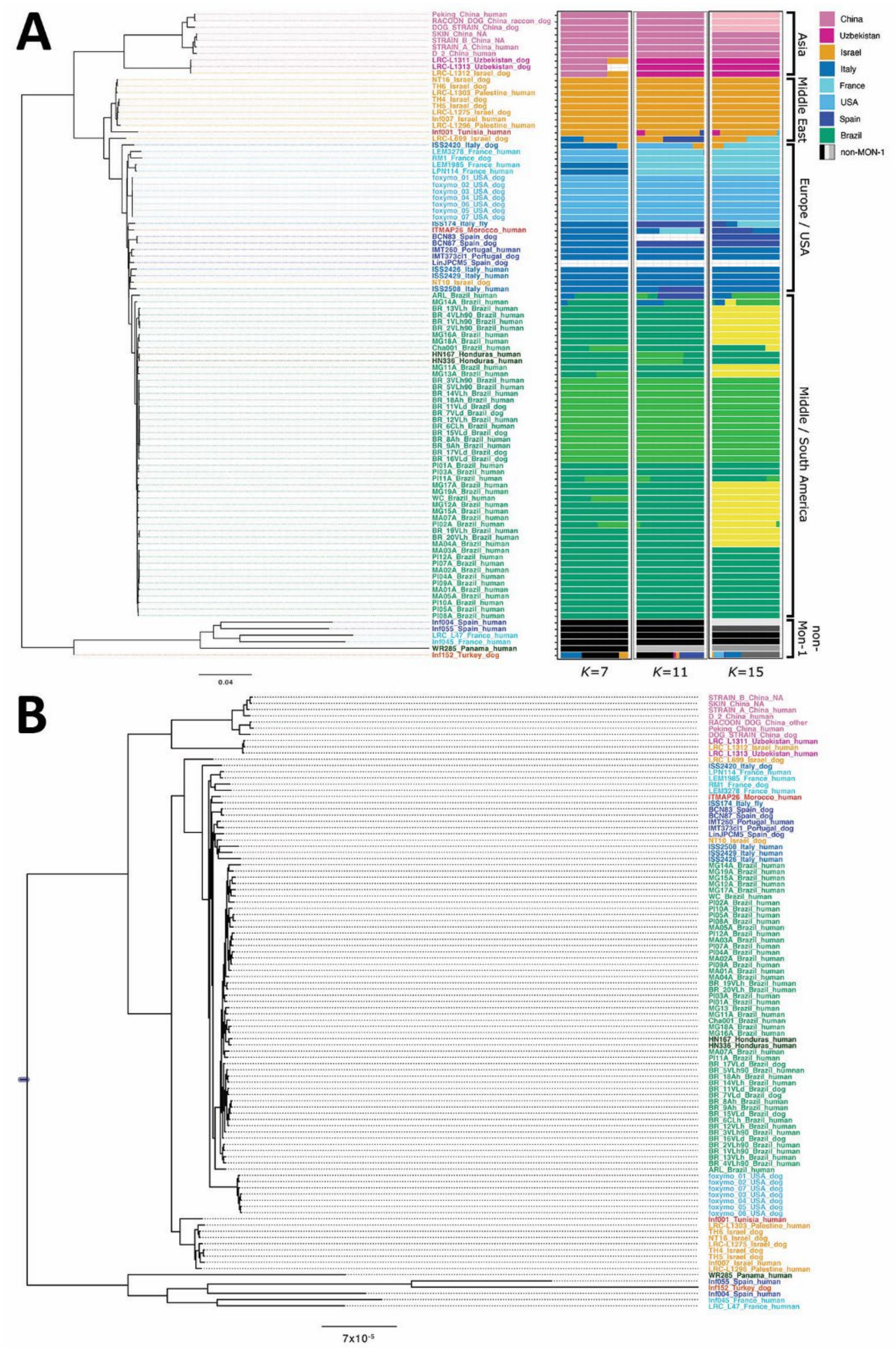

Appendix 2 Figure 2. Evolutionary origin of US hound *Leishmania* isolates in the broader context of *L. infantum* isolates. Phylogenies were reconstructed based on whole-genome genotype calls of 99 parasite samples presenting the dominant *L. infantum* zymodeme MON-1 from the United States, Europe, South America, the Middle East, and China, as well as non-MON-1 isolates from Europe. A) Neighbor-joining tree based on pairwise Nei's distances. The 3 rightmost columns indicate population grouping using admixture with best fitting total number of groups (Appendix 2 Figure 1, panel B). B) Maximum-likelihood tree for the same sample set.

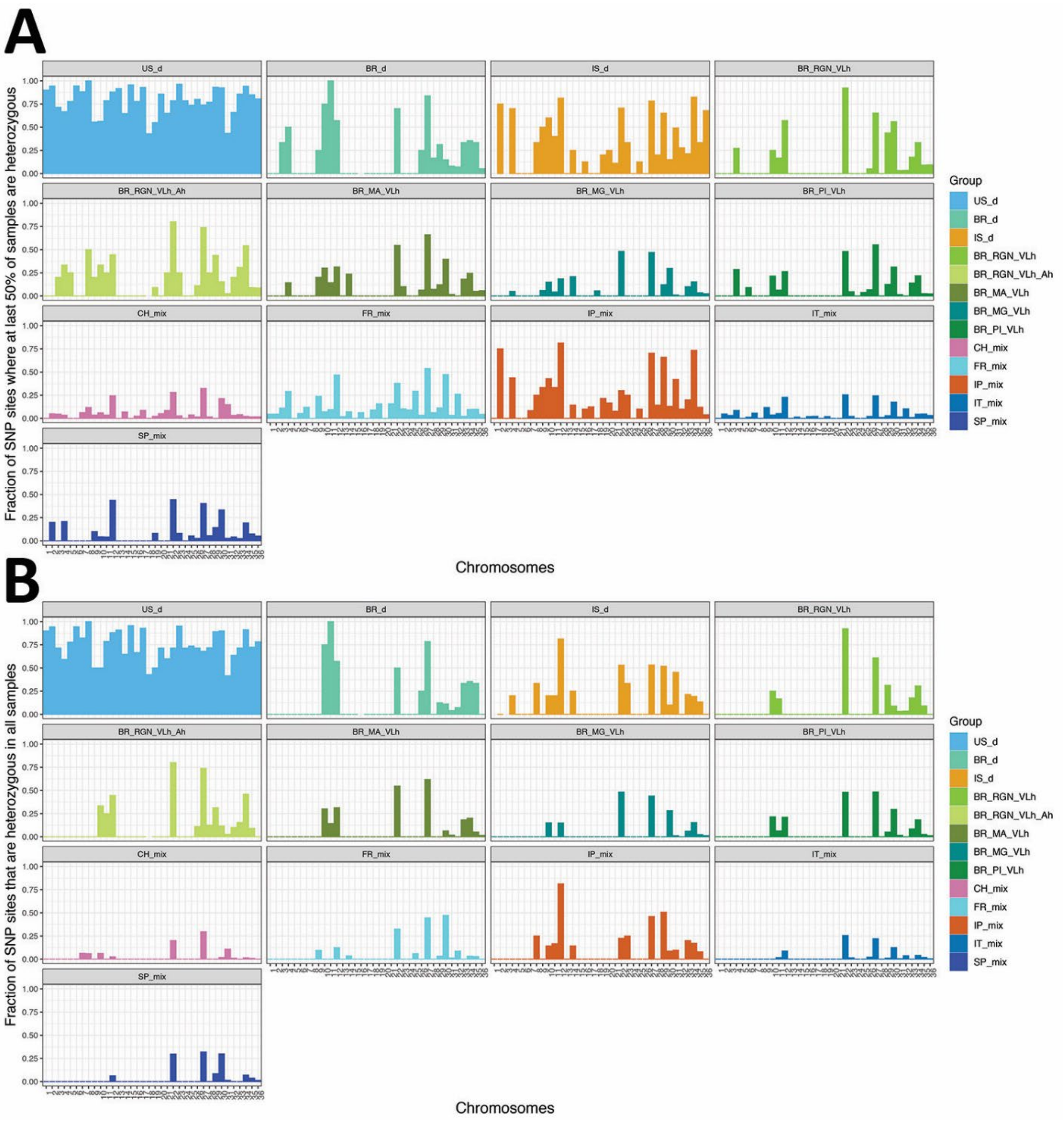

Appendix 2 Figure 3. High fraction of shared heterozygous sites in *Leishmania* isolates from US hunting hounds. Shown are fractions of sites which are heterozygous shown by chromosome and group. A) Fraction of sites for which at least 50% of samples are heterozygous. B) Fraction of sites for which all samples are heterozygous.

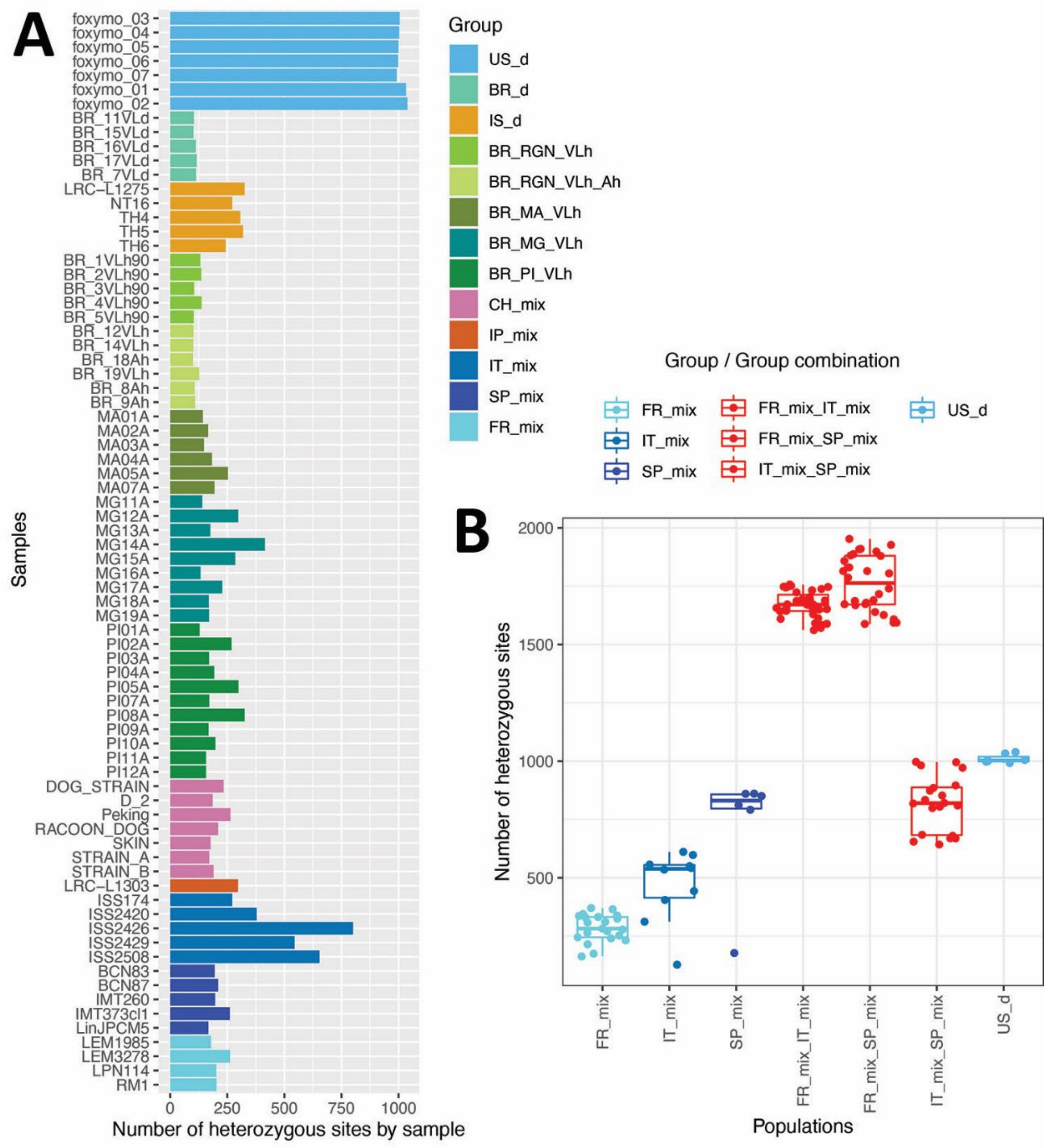

Appendix 2 Figure 4. High levels of sample heterozygosity of US samples and putative hybrid origin. A) Number of heterozygous sites for each sample from the different populations specified in Table 1. For the US population with a total of 1,182 segregating sites, 79% of them are heterozygous sites present across all 7 samples. A total of 88% of all SNP sites are called as heterozygote in at least 2 US samples. B) Comparison of number of heterozygous sites in the samples from the U.S. (US_d) in contrast to artificially constructed hybrids within European populations (FR_mix, IT_mix, SP_mix) or between European populations (FR_mix_IT_mix, FR_mix_SP_mix, IT_mix_SP_mix). Hybrids have been generated by generating one artificial hybrid for each sample by randomly choosing 1 of 2 alleles for each heterozygous position and paring all such generated haplotypes in all possible combinations either with 1 or between 2 European populations.

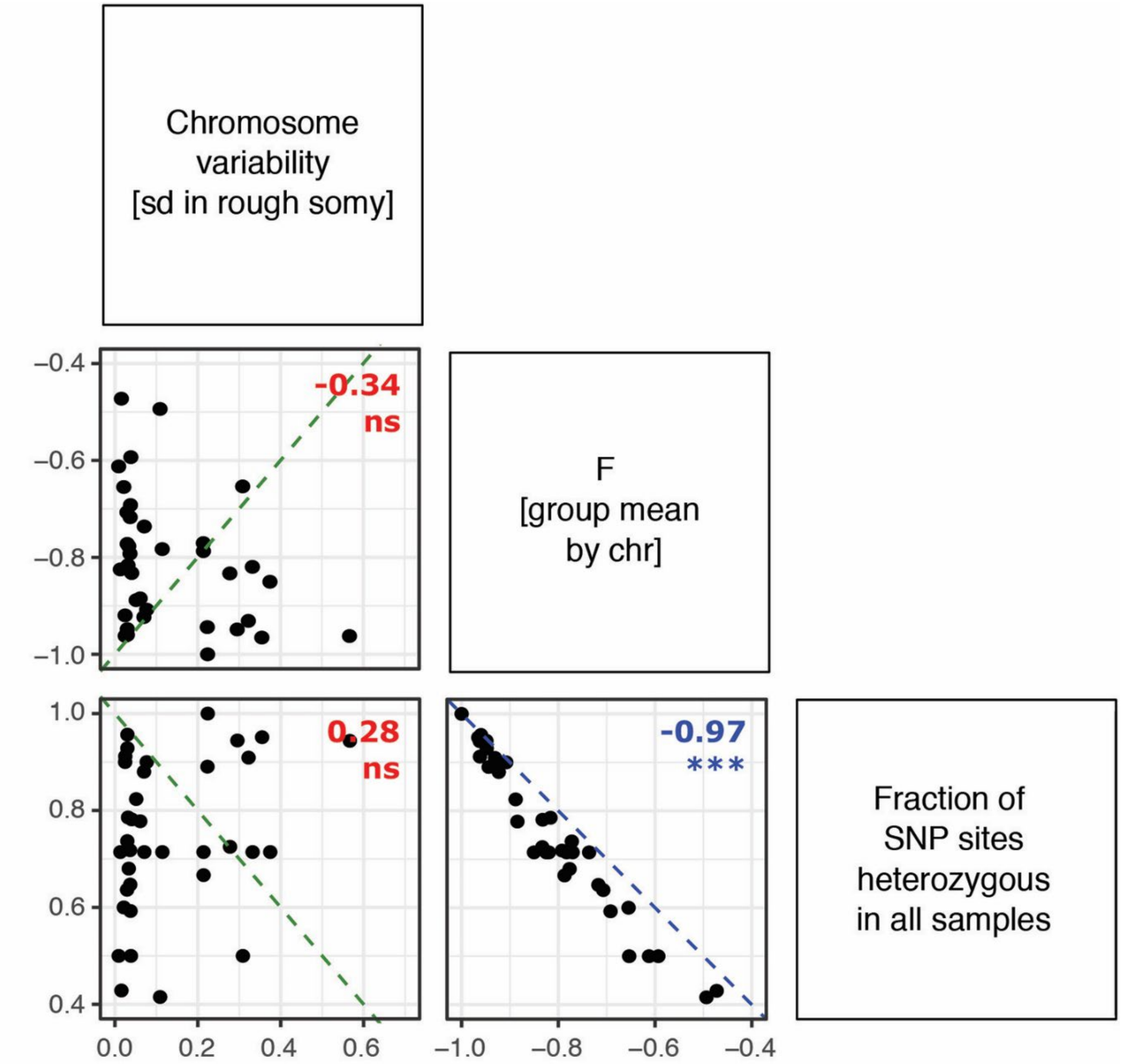

Appendix 2 Figure 5. No correlation between somy variability and heterozygosity per chromosome in *Leishmania* from US hounds. Dashed green lines indicate the expected correlation between somy variability and observed heterozygosity if changes in somy—captured as somy variability—contributed to reduction in heterozygosity in this population. The dashed blue line shows the expected correlation between 2 different measures for heterozygosity. Adjusted p-values (FDR) are indicated by ns (not significant, FDR>0.05) and *** (FDR<0.001).

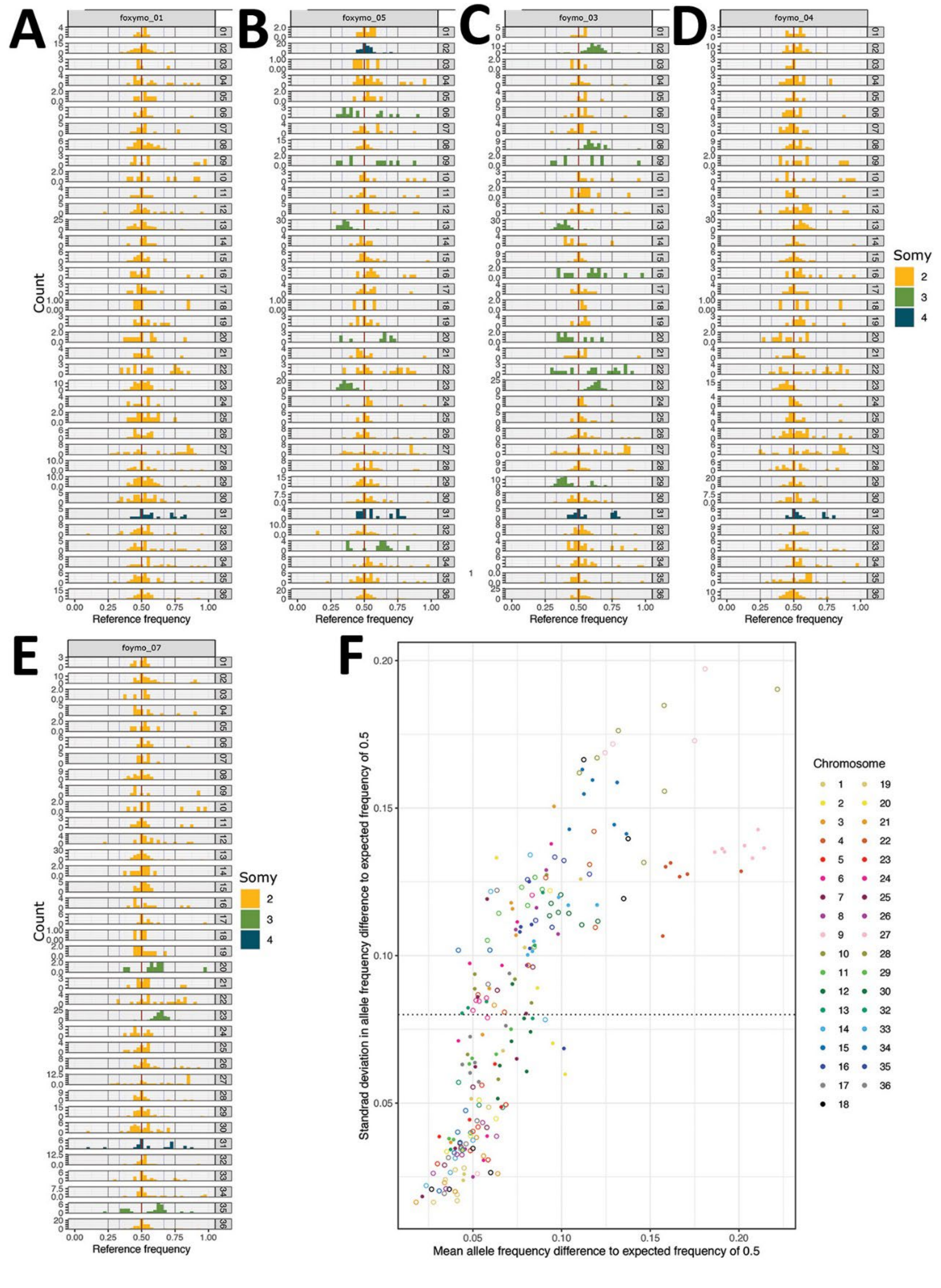

Appendix 2 Figure 6. Evaluation of within-isolate diversity though allele frequencies. Sample-specific allele frequency distributions agree with coverage-based somy estimates. A-E) For 5 out of the 7 US hound–derived isolates chromosome-specific allele frequencies are shown for polymorphic sites in the respective isolate, estimated from quality-filtered sequencing coverage for each allele. Histograms are

colored based on somy estimated from sequencing coverage. Vertical lines indicate frequencies of 0.5 (red), 1/3 and 2/3 (blue) and 1/4 and 3/4 (black) to indicate where peaks of allele frequency distributions should be located given different somies. F) Mean deviation of SNP allele frequencies from the expected frequency of 0.5 for all diploid chromosomes versus standard deviation of these deviations. Dotted lines are drawn thresholds based on a previous classification, with suspected multiclonal isolates being above the horizontal line (*3*). For most chromosomes the allele frequencies match those expected from chromosome somies, although chromosomes 4, 9, 10, 16, 22, 27, and 34 show somewhat larger deviations, which could suggest copy number changes in a subset of cells.
